# LpCat1 Promotes Malignant Transformation of Hepatocellular Carcinoma Cells by Directly Suppressing STAT1

**DOI:** 10.3389/fonc.2021.678714

**Published:** 2021-06-04

**Authors:** Weidan Ji, Zhangxiao Peng, Bin Sun, Lei Chen, Qin Zhang, Minggao Guo, Changqing Su

**Affiliations:** ^1^ Department of Molecular Oncology, Eastern Hepatobiliary Surgical Hospital & National Centre for Liver Cancer, Navy Military Medical University, Shanghai, China; ^2^ Department of General Surgery, Shanghai Sixth People’s Hospital, Shanghai Jiaotong University School of Medicine, Shanghai, China

**Keywords:** LpCat1, STAT1, cell cycle, proliferation, metastasis, hepatocellular carcinoma (HCC)

## Abstract

Hepatocellular carcinoma (HCC) is a malignant cancer with rapid proliferation and high metastasis ability. To explore the crucial genes that maintain the aggressive behaviors of cancer cells is very important for clinical gene therapy of HCC. LpCat1 was reported to be highly expressed and exert pro-tumorigenic effect in a variety of cancers, including HCC. However, its detailed molecular mechanism remained unclear. In this study, we confirmed that LpCat1 was up-regulated in HCC tissues and cancer cell lines. The overexpressed LpCat1 promoted the proliferation, migration and invasion of HCC cells, and accelerated cell cycle progression, while knocking down LpCat1 significantly inhibited cell proliferation, migration and invasion *in vitro* and *in vivo*, and arrested HCC cells at G0/G1 phase. Moreover, we proved for the first time that LpCat1 directly interacted with STAT1 which was generally recognized as a tumor suppressor in HCC. High levels of LpCat1 in HCC could inhibit STAT1 expression, up-regulate CyclinD1, CyclinE, CDK4 and MMP-9, and decrease p27kip1 to promote cancer progression. Conversely, down-regulation of LpCat1 would cause the opposite changes to repress the viability and motility of HCC cells. Consequently, we concluded that LpCat1 was a contributor to progression and metastasis of HCC by interacting with STAT1.

## Introduction

Hepatocellular carcinoma (HCC) is one of the most common malignant tumors. Many factors, such as activation of oncogenes and inactivation of tumor suppressor genes, could cause unrestricted growth of HCC cells. Among them, hundreds of cancer-associated genes are closely related to the occurrence and development of HCC. With the development of a variety of “omics” technologies such as genomics, proteomics and transcriptomics, accumulating molecular markers related to malignant transformation of HCC cells have been discovered. Therefore, further study of HCC-related genes and identifying novel treatment targets have become an urgent task.

LpCat1 (lysophosphatidylcholine acyltransferase 1) is a key enzyme in phospholipid metabolism, which is responsible for promoting the synthesis and remodeling of phosphatidylcholine (PC) (1, 2). As the main component of cell membranes, PC plays an important role in maintaining cell structure and biological functions (3, 4). Previous studies have been reported that PC was involved in the malignant progression of many cancers (5). The expression of LpCat1 was obviously increased in various tumors including prostate cancer, gastric cancer and breast cancer (6–8). Its high expression is positively correlated with the development of malignant tumors. Emerging evidence showed that up-regulation of LpCat1 promotes cancer cell proliferation and metastasis, while knocking down LpCat1 could inhibit growth of cancer cells by inducing cell cycle arrest at G0/G1 phase, which may serve as a novel target of aggressive progression in oral squamous cell carcinoma (OSCC), renal clear cell carcinoma and lung adenocarcinoma (9–11). In HCC cells, LpCat1 overexpression enriched PCs and promoted cell proliferation, migration, and invasion (12). However, the detailed molecular mechanism remains poorly understood.

In the present study, we firstly confirmed that LpCat1 was overexpressed in the tissues and cells of HCC. Furthermore, we investigated the functional effect of up- or down-regulation of LpCat1 on the proliferation, metastasis and cell cycle of HCC cells. Based on identification of LpCat1 interactors by mass spectrometry, we confirmed that LpCat1 could directly interact with STAT1. As we known, STAT1 is generally recognized as a tumor suppressor gene in cancer cells (13–15). Our data suggested that LpCat1 might promote the progression and metastasis of HCC by directly interacting with STAT1.

## Materials and Methods

### Human HCC Tissue Samples and Cell Lines

The clinical tissue samples were collected from HCC patients who underwent radical resection in our hospital. Informed consents have been signed for all the patients. Tissue microarray including 90 pairs of HCC tissue samples (including paired tumorous tissues and adjacent liver tissues) was detected by immunohistochemistry. Human HCC cell lines Huh7, Hep3B, HepG2, HCCLM3 and immortalized normal liver cell L02 were purchased from Chinese Academy of Sciences Cell Bank (Shanghai, China). The cells were cultured in DMEM medium with 10% FBS (Gibco, USA), 100 units/mL penicillin, 100 μg/mL streptomycin (Invitrogen, USA) and incubated in a CO_2_ incubator at 37°C.

### Establishment of Stable LpCat1 Overexpressing and Knockdown HCC Cell Sublines

LpCat1 was stably overexpressed and knocked down in the human HCC cell lines Hep3B and Huh7 by means of lentiviral approach. Briefly, lentiviral-mediated LpCat1 overexpression vector (LpCat1 OV) and the null control vector (O Control) were constructed by inserting the coding sequence of LpCat1 (Gene ID: 79888) or nonsense sequence into lentivirus vector. The shRNA1, shRNA2, shRNA3 of LpCat1 and scramble shRNA were designed and constructed into lentivirus shRNA knockdown vectors. The sequences of three shRNAs were shown in **Supplementary Table 1**. According to knockdown effect of LpCat1 confirmed by western blot and qRT-PCR, shRNA1 of LpCat1 (LpCat1 KD) was chose for subsequent experiments. The scramble shRNA (K Control) was used as control. All lentivirus vectors carried U6 promoter, EGFP gene and puromycin resistance gene, cellular immunofluorescence was used to observe EGFP positive HCC cells at 72 h after infection (**Supplementary Figure 1**). The stably transfected cells selected by puromycin were used for all the *in vitro* and *in vivo* experiments.

### RNA Extraction and Quantitative Polymerase Chain Reaction (qPCR)

Total RNA from HCC tissues (n=40) and cell lines were extracted by using TRIzol reagent. Complementary DNA (cDNA) was synthesized from 1 μg total RNA using Eastep RT Master Mix Kit (Promega, shanghai, China), and PCR was performed using Eastep qPCR Master Mix Kit (Promega, shanghai, China). PCR was run on the StepOne Plus Real-Time PCR System (Applied Biosystems, Foster City, CA, USA), and the transcript amounts for the target genes were estimated from the respective standard curves and normalized to the glyceraldehyde-3-phospate dehydrogenase (GAPDH). The primer sequences involved in this study were shown in **Supplementary Table 2**.

### Western Blotting and Immunohistochemistry (IHC)

For western blotting, HCC cells were lysed with RIPA lysis buffer (1% PMSF, 1% phosphatase inhibitor and 1% protease inhibitor were added). The antibody information was shown in **Supplementary Table 3**.

For IHC, the tissue sections were incubated with the anti-LpCat1 antibody (Abcam) at 4°C overnight. Negative controls were performed by replacing the primary antibody with PBS. Then the sections were incubated with the horseradish peroxidase (HRP)-conjugated secondary antibody (Maixin-Bio, Fuzhou, China) at room temperature for 1 h, and the immunoreactivity was detected utilizing diaminobenzidine (DAB) substrate (Maixin-Bio). Counter-staining of the nucleus was performed using hematoxylin. The assessment of the staining was conducted by two investigators independently.

A quick scoring system that is the product of the intensity and percentage of the positive signal was used to calculate the LpCat1 IHC scores. Briefly, a signal of 0 indicated no staining, 1 indicated weak staining, 2 indicated intermediate staining and 3 indicated strong staining. Percentage scores were assigned as follows: 0 corresponded to 0%, 1 to 1-25%, 2 to 26-50%, 3 to 51-75%, and 4 to > 75%. The product of two terms rangs from 0 to 12, and the median value of total staining scores was identified as the optimal cut-off value. If the evaluated score was lower than the median, the indicator expression of in those HCC samples was classified as low; otherwise, it was classified as high (16).

### Cell Proliferation and Clone Formation Assays

CCK8 assay was carried out to detect the proliferation ability of different experimental groups *in vitro*. Clone formation assay was used to evaluate the clone formation ability *in vitro.* The specific experimental procedures were carried out as previously described (17). All experiments were repeated at least three times.

### Migration and Invasion Assay

*In vitro* cell migration and invasive abilities were measured by transwell assay. In brief, totally 3 × 10^4^ cells were added onto the upper chamber, uncoated for migration assay, or coated with a thin layer of BD Matrigel Matrix (BD Biocsciences, MD) for invasion assay. After culture for 48 h, the migrated and invaded cells were fixed with 4% formaldehyde and subsequently stained with 0.1% crystal for assessment. For each experimental group, five random fields of stained cells were photographed and counted. All experiments were repeated at least three times.

### Wound-Healing Assay

*In vitro* cell mobility was evaluated by wound-healing assay. In brief, cells were seeded in 6-well plate. The scratches were observed and images were captured with a microscope at 0 and 72 h pro-injury. Image-J software was used to determine the average area.

### Cell Cycle

For cell cycle analysis, cells (1×10^6^ cells per well) were seeded into 6-well plates, cultured for 48 h, washed twice with cold PBS, stained with propidium iodide (PI) and Annexin V, then examined cell cycle by flow cytometer (FACS420, BD Biosciences, San Jose, CA) within 2 h.

### Immunoprecipitation Tandem Mass Spectrometry (IP-MS) and Co-IP Assay

To define the proteins which interacted with LpCat1, we performed IP-MS assay. Proteins of Huh7 cells stably transfected with LpCat1 overexpressed and null control vectors were collected. Immunoprecipitations was done by incubating the cell lysate (1 mg) with the anti-LpCat1 antibody (1μg) overnight at 4°C. Protein A/G Sepharose beads (Santa Cruz Biotechnology) were added to the lysate antibody solution for 2 h of incubation. Then, beads were washed and boiled in 5× SDS sample buffer. IPs for mass spectrometry were separated by SDS-PAGE and coomassie blue stained, the bands were retrieved and analyzed by MS. Liquid chromatography/mass spectrometry (LC/MS) was performed on a Thermo ScientificTM Q Exactive Plus coupled to an ultra-high performance liquid chromatography unit (Thermo Scientific Dionex Ultimate 3000) *via* a Nanospray Flex Ion Source (Thermo Fisher Scientific).

Co-IP assay was performed to verify the interaction between LpCat1 and STAT1. HCC cells with LpCat1 overexpressed and negative control vector were lysed in IP lysis buffer (#P0013, Beyotime Biotechnology). Then the lysates were incubated with the anti-LpCat1 and anti-STAT1 antibodies separately overnight at 4°C. Protein A/G-agarose beads were added for 2 h. The beads were collected and washed with lysis buffer for three times. The precipitated proteins were eluted and denatured in 5 × SDS loading buffer and analyzed by western blotting.

### The Xenograft Assay and Metastasis Assay *In Vivo*

The xenograft model and metastasis model were established by implantation of stably-transfected Hep3B cell lines. Mice were handled and housed according to protocols approved by the Animal Ethics Committee of Navy Military Medical University (Shanghai, China).

For *in vivo* metastasis assay, 1 × 10^6^ Hep3B cells stably transfected with different constructs were suspended in 100 μL of serum-free DMEM per male BALB/c-nu/nu mice, and injected into nude mice through the tail vein (6 mice per group). After four weeks, mice were sacrificed, and their lungs were dissected. Lung metastasis was diagnosed visual inspection and confirmed by histological staining.

For the xenograft model, 1 × 10^7^ Hep3B cells of different groups were subcutaneously injected into left flank of 4-week-old male mice, respectively. Two month later, the subcutaneous tumor was removed and cut into pieces of the same size as 1 mm^3^. Twenty-four male BALB/C nude mice at 4-week-old (Shanghai SLAC Laboratory Animal Center of Chinese Academy of Sciences, Shanghai, China) were randomly divided into four groups (n = 6 per group) and the tumor pieces were implanted into the livers of each group to mimic the primary HCC. Six weeks later, mice were sacrificed and the tumors were harvested. Tumor weight was weighed, and tumor volume was calculated using the formula ‘‘*a*×*b* ^2^×0.5’’, in which *a* and *b* represent the maximal and minimal diameters. The tumor specimens were fixed with phosphate-buffered neutral formalin, and prepared for examining the expression of LpCat1, STAT1, CyclinD1, ki67 and MMP-9 by immunohistochemical analysis.

### Statistical Analysis

The results were presented as mean ± standard deviation (SD). Statistical evaluation of the data was performed with one-way ANOVA. Comparisons between two groups were made by using the paired *t* test. The P value less than 0.05 was considered statistically significant. All the statistical analyses were analyzed with SPSS version 19.0 software.

## Results

### LpCat1 Was Frequently Up-Regulated In Human HCC Tissues and HCC Cell Lines

**Figure 1:**
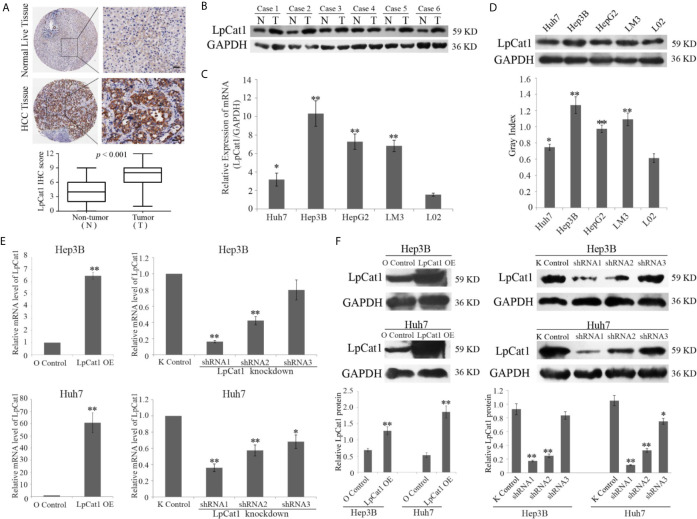
LpCat1 was frequently up-regulated in human HCC tissues and HCC cell lines. **(A)** The expression level of LpCat1 was detected by immunohistochemical (IHC) analysis in HCC microarray. The representative images of LpCat1 expression in HCC tissues and corresponding normal live tissues by IHC were taken (scale bar = 50 µm). **(B)** Western blot analysis was used to evaluate the expression of LpCat1 in 6 paired human HCC tissues and adjacent normal tissues. N and C represented adjacent normal parenchyma and cancer tissue, respectively. GAPDH was used as an internal standard. **(C, D)** The expression of LpCat1 in HCC cell lines was detected by qRT-PCR and western blotting. Data were expressed as mean±SD, *p < 0.05, **p < 0.001. **(E, F)** qRT-PCR and western blot analysis of LpCat1 levels in Hep3B and Huh7 cells stably transfected with LpCat1-experessed or three shLpCat1 lentivirus vectors. Data were expressed as mean±SD, *p < 0.05, **p < 0.001.

We examined 90 pairs of clinical HCC tissues by IHC, and found the expression of LpCat1 was dramatically elevated in HCC tissues, comparing with the para-cancerous tissues (**Figure 1A**). Correspondingly, LpCat1 was proved to be also up-regulated in HCC tissues by qRT-PCR and western blotting assay (**Figure 1B**, **Supplementary Figure 2**). After statistical analysis, the LpCat1 IHC scores in HCC tissues were obviously higher than those in normal liver tissues. 63 of 90 (70%) HCC samples were considered as LpCat1-high expression group. We then analyzed the correlation between LpCat1 protein expression and the clinicopathological characteristics of the patients with HCC (**Supplementary Table 4**). Among the clinical classifications, high expression level of LpCat1 in HCC tissues were significantly correlated with vascular invasion (p = 0.002) and TNM stage (p = 0.015). The results indicated that abnormal expression level of LpCat1 may be associated with the progression and metastasis of HCC.

In addition, we detected the mRNA and protein expression levels of LpCat1 in four HCC cell lines and normal liver cell line L02. Similarly, compared to normal liver cells, LpCat1 is generally highly expressed in HCC cell lines (**Figures 1C, D**). Hep3B and Huh7 cell lines were chose for subsequent experiments. In order to artificially change the expression status of LpCat1, we designed the overexpression and knockdown vectors for LpCat1 and packaged them into lentivirus. Through multiple screening in the *in vitro* experiments, Hep3B and Huh7 cells with LpCat1 overexpression and knockdown stable transgenic cell sublines were successfully constructed. Efficient depletion or over-expression was evaluated by qRT-PCR and western blot (**Figures 1E, F**). We chose HCC cells transduced with lentivirus bearing LpCat1 to over-expression and shRNA1 to knockdown LpCat1 expression for subsequent studies.

### LpCat1 Promoted The Proliferation, Migration and Invasion of HCC Cells

**Figure 2:**
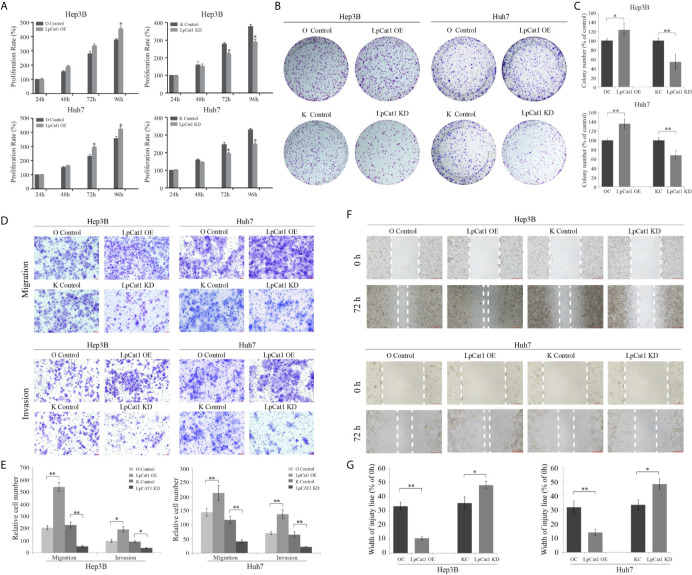
LpCat1 promoted the proliferation, migration and invasion of HCC cells. **(A)** The proliferative ability of Hep3B and Huh7 cells after stably transfection was evaluated by CCK8 assay. **(B, C)** Neoplastic capacity of Hep3B and Huh7 cells transfected with over-expression or knockdown vectors of LpCat1 was assessed by plate colony formation assay. Cell colonies were counted and analyzed, then showed in histogram. **(D, E)** Representative images and quantification of transwell assays indicated the migration and invasive capability of HCC cells stably transfected with LpCat1 over-expression or knockdown vectors. The penetrated cells were counted and analyzed, and showed in histogram. **(F, G)** Wound-healing assays were performed to assess HCC cells migration ability. Wound closure was determined 72 h after the scratch. Data were presented as mean±SD, *p < 0.05, **p < 0.001.

To confirm the effect of LpCat1 expression on the proliferation of HCC cell lines, we used HCC stably transgenic cell sublines with LpCat1 overexpression or knockdown to perform CCK8 and clone formation assays. The data indicated that the up-regulation of LpCat1 obviously promoted the proliferation of HCC cells compared to the control cells. The proliferation rate was significantly inhibited at 72 h and 96 h in HCC cells with LpCat1 knockdown (**Figure 2A**). The clone formation assay showed that the number and size of clones of HCC cells were increased in the LpCat1 over-expression group and decreased after knocking down LpCat1 (**Figures 2B, C**). In summary, LpCat1 plays a key role in maintaining the unlimited proliferation of HCC cells.

**Figure 3:**
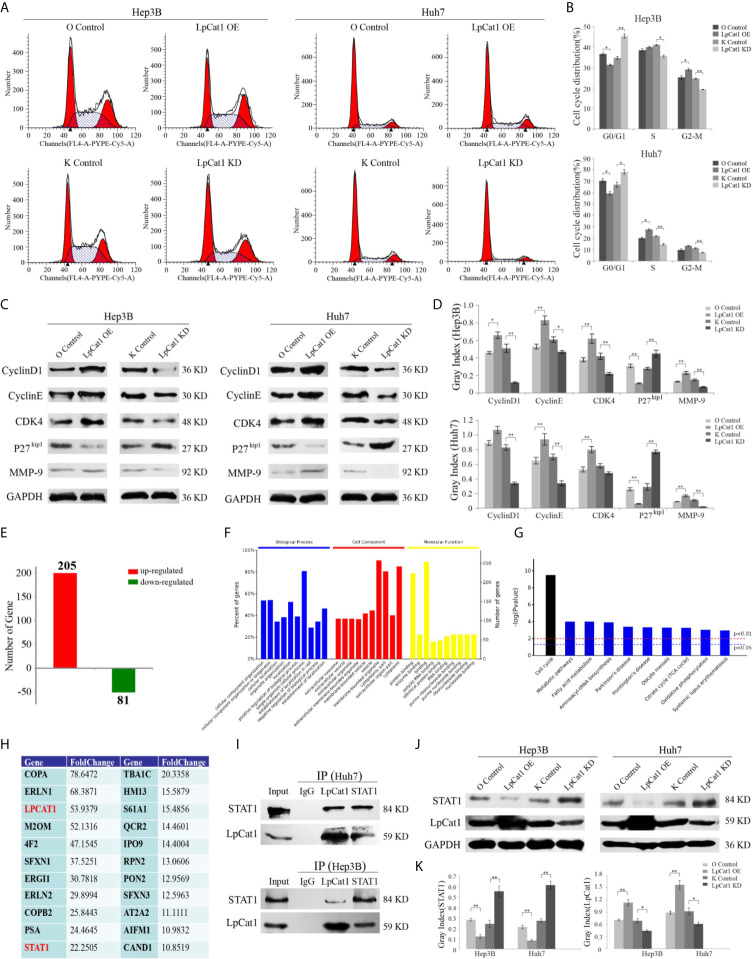
LpCat1 interacted with STAT1 and facilitates cell cycle progression. **(A, B)** Flow cytometry analysis of cell cycle in the LpCat1 over-expression and knockdown HCC cells. Data of cell cycle were shown in histograms. Data were presented as mean±SD, *p < 0.05, **p < 0.001. **(C, D)** The expression of cell cycle related proteins was detected by western blotting in HCC cell lines with LpCat1 stably over-expression or knockdown. GAPDH was used as loading control, and bands were semi-quantitatively analyzed by using Image J software, normalized to GAPDH density. Data were presented as mean±SD, *p < 0.05, **p < 0.001. **(E)** IP-MS analysis of LpCat1-binding proteins. The proteins of LpCat1 OE and OC groups in Huh7 cells were obtained and performed IPs with anti-LpCat1 antibody. The differential proteins identified by MS analysis between two groups were counted, and the number of genes corresponding to differential proteins was showed in histogram. **(F)** All these 286 genes were subject to GO term analysis. **(G)** Enriched KEGG pathway analysis of 286 genes by DAVID. **(H)** Analyses of identified proteins precipitated with the LpCat1 antibody (Fold change >10). **(I)** Co-IP analysis indicated the endogenous interaction between LpCat1 and STAT1 proteins in HCC cell lines. **(J, K)** Western blotting analysis of STAT1 protein in HCC cell lines with LpCat1 stably over-expression or knockdown. GAPDH was used as internal control. Data were presented as mean±SD, *p < 0.05, **p < 0.001.

Transwell and wound-healing assays were carried out to explore the effect of LpCat1 expression on the migration and invasion of HCC cells. In the LpCat1 over-expression group, the number of penetrating cells were markedly elevated compared with the control cells (**Figures 2D, E**), and the wound closed faster than the control cells at 72 h after scratching (**Figures 2F, G**). Inversely, the number of penetrating shLpCat1-transfected HCC cells was decreased compared with the shMock-transfected cells. The wounds in the shLpCat1-transfected cells closed later than the shMock-transfected cells. Moreover, the expression level of MMP-9 was detected in HCC cell lines with different levels of LpCat1 (**Figure 3C**). Consistent with the above experimental results, the expression of MMP-9 was positively correlated with LpCat1. Over-expression of LpCat1 enhanced HCC cell mobility accompanied by elevated expression of MMP-9, and vice versa. Therefore, the high expression of LpCat1 contributes to the increased migration and invasion ability of HCC cells.

### LpCat1 Facilitated Cell Cycle Progression *via* Up-Regulation of Cell Cycle Related Proteins

In order to evaluate the influence of LpCat1 expression on the cell cycle of HCC cells, we detected the cell cycle distribution of LpCat1 over-expressed or knocked-down cells by flow cytometry (**Figures 3A, B**). The results showed that comparing with the knockdown control group, the percentages of G0/G1 phase were increased by 10.66% and 11.18% in the LpCat1 knockdown groups of Hep3B and Huh7 cells, respectively. In the LpCat1 overexpression groups, the percentages of G0/G1-phase were significantly reduced by 6.26% and 11.03%, with the number of cells entering the S phase and G2-M phase increased. These results suggested that the high level of LpCat1 contributed cell cycle progression, inhibition of LpCat1 expression arrested cell cycle at G0/G1 phase.

Taken together, LpCat1 regulates the proliferation and cell cycle of HCC cells, but the accurate molecular mechanism is still unclear. We examined the effect of LpCat1 on cell cycle regulators in HCC cells (**Figures 3C, D**). Western blotting results indicated that the over-expression of LpCat1 up-regulated the expression of CyclinD1, CyclinE and CDK4, and their expression was dramatically decreased in the HCC cells with LpCat1 knockdown. p27^kip1^ was reported to be a cell cycle inhibitor in HCC (18). Moreover, we found the expression of p27^kip1^ is negatively correlated with LpCat1. Thus, induction of LpCat1 overexpression up-regulated CyclinD1, CyclinE and CDK4, but down-regulated p27^kip1^ in HCC cells. In contrast, the expression of CyclinD1, CyclinE and CDK4 was decreased and p27^kip1^ was activated in HCC cells with LpCat1 depletion. Accordingly, LpCat1 influences cell cycle to promote HCC proliferation by activating cell cycle-related genes.

### LpCat1 Restrained STAT1 Expression by Directly Interacting With STAT1

To explore the proteins interacting with LpCat1, we used the LpCat1 antibody to perform immunoprecipitation (IP) assay and identified targets by mass spectrometry (MS) technology. Totally 286 proteins with abnormal expression were screened out in the LpCat1-overexpressed HCC cells, comparing with the control group of Huh7 cells. Among them, 205 proteins were up-regulated, and 81 proteins were down-regulated (**Figure 3E**). Then, we analyzed the functions of these 286 dys-regulated genes by using DAVID (The Database for Annotation, Visualization and Integrated Discovery). The top 10 GO terms related to “biological processes”, “molecular functions” and “biological cellular components” are shown in **Figure 3F**. The predominantly enriched biological process was “single-organism cellular process”. In terms of cellular components, the “membrane-bounded organelle and cytoplasm” biological processes were the most-enriched subcategories. These genes were also enriched in molecular functions included “protein binding”. KEGG pathway analysis showed “cell cycle” was the most enriched subcategories (**Figure 3G**). Proteins precipitated with the LpCat1 antibody more than 10-fold up-regulation in the LpCat1-overexpressed Huh7 cells were listed in **Figure 3H**. Among them, STAT1 has been reported to be closely related to the growth and cell cycle of HCC cells. Combining the data of KEGG pathway analysis and cell cycle detection, we speculated that LpCat1 accelerated HCC progression by interaction with STAT1. The direct interaction between LpCat1 and STAT1 was proved by co-IP and western blotting assays in Hep3B and Huh7 cells (**Figure 3I**).

Based on the interplay of LpCat1 and STAT1, we found the expression of LpCat1 in HCC cells and tissues is significantly negatively correlated with STAT1 (**Figures 3J, K**, **Supplementary Figure 3**). As a tumor suppressor gene, STAT1 could inhibit the growth of HCC and induce cell arrest at G0/G1 phase (19). The generally high level of LpCat1 would inhibit the tumor suppressor STAT1, thereby accelerating cell cycle and proliferation of HCC cells.

### LpCat1 Promoted Tumorigenesis and Metastasis of HCC *In Vivo*

**Figure 4:**
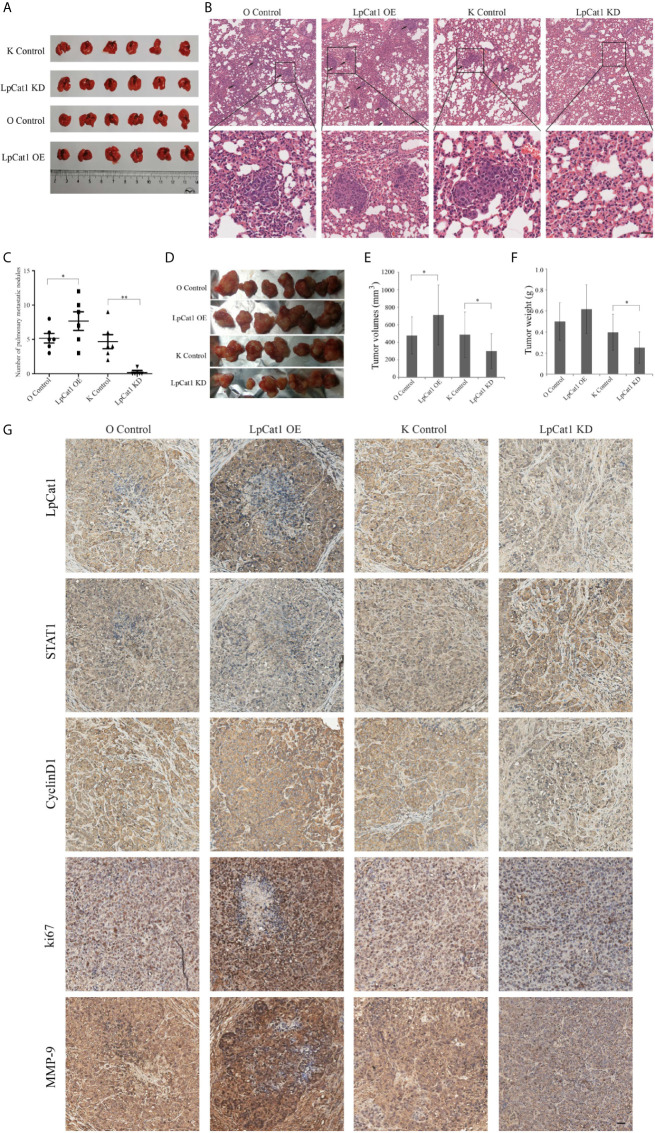
LpCat1 promoted tumorigenesis and metastasis *in vivo*. **(A)** Hep3B cells stably transfected with different constructs were separately injected into the tail vein of nude mice to form metastasis model. The photo of lungs in each group (n = 6 mice per group) was taken. **(B)** HE staining showed the pulmonary metastatic nodules of nude mice (scale bar = 50 µm). **(C)** The numbers of metastatic nodules in the lungs of each mouse were counted and analyzed. The data were expressed as the mean ± SD, *P < 0.05, **P < 0.001. **(D)** Effect of LpCat1 on liver orthotopic xenograft tumor growth in nude mice. After the observation, mice were sacrificed and the tumors were harvested. **(E, F)** Tumor volume was calculated and tumor weight was weighed. The data were expressed as the mean ± SD, *P < 0.05. **(G)** Immunohistochemical examination of the indicated factor expression in the xenograft tumors (scale bar = 50 µm).

In order to examine the effect of LpCat1 on tumor metastasis *in vivo*, we established metastasis model by implantation of lentivirus-mediated stably transfected Hep3B cells with up- or down-regulation of LpCat1 expression by tail vein injection. Four weeks after injection, the number of lung nodules in the mice of LpCat1 overexpression group was significantly greater than the control group. In the LpCat1 knockdown group of mice, the number of lung nodules was markedly reduced or even absent, which implicated down-regulation of LpCat1 expression could effectively inhibit metastasis of HCC cells *in vivo* (**Figures 4A–C**).

In addition, the xenograft model was established to evaluate the effect of LpCat1 on tumor growth. Consistent with the *in vitro* experimental data, LpCat1 enhanced tumorigenicity of HCC cells *in vivo* (**Figure 4D**). Compared with the control group, the tumor volume and weight of the LpCat1 overexpression group significantly increased. The tumor growth was substantially inhibited in the LpCat1 depletion group, as shown by smaller tumor size and lighter tumor weight (**Figures 4E, F**). Moreover, we detected the expression of LpCat1, STAT1, CyclinD1, ki67 and MMP-9 in the tumor tissues by immunohistochemistry (**Figure 4G**). In the LpCat1 up-regulated group, the expression of STAT1 was reduced, and the expression of ki67, MMP-9 and CyclinD1 was positively increased, compared with the control group. Conversely, down-regulation of LpCat1 promoted the expression of STAT1, and suppressed the expression of ki67, MMP-9 and CyclinD1. These results suggested that LpCat1 could promote the tumorigenesis and metastasis of HCC *in vivo*.

## Discussion

The development of HCC was a complex process involving multiple genes. Identifying the key genes maintaining the malignant phenotype would be of great significant for molecular targeted therapy of HCC. Abnormally active of lipid synthesis was observed in HCC pathophysiology (20). However, it was still unclear how these lipid synthesis enzymes contribute to the progression of HCC. Importantly, LpCat1 is an important enzyme responsible for lipid synthesis, which mainly catalyzes the conversion of lysophosphatidylcholine (LPC) into phosphatidylcholine (PC) (21, 22). The molecular lipidomic study of clinical HCC specimens reported that PC species increased in HCC tissues with reduction of LPC, which indicated LpCat1 is closely related to the development of HCC (12). Genomic analysis of LpCat1 copy number in TCGA database revealed LpCat1 gene amplification exists in various tumors, especially lung adenocarcinoma (18%, 40/230), lung squamous cell carcinoma (19%, 94/501), ovarian serous cystadenocarcinoma (14%, 79/579), esophageal carcinoma (13%, 23/184) and so on (23–25). LpCat1 was generally recognized as an oncogene due to its up-regulation in a variety of tumor tissues including HCC. In our study, we firstly confirmed that LpCat1 was overexpressed in HCC tissues and HCC cells lines. LpCat1 protein expression level in HCC patients was significantly correlated with the vascular invasion and the TNM stage. Our experimental results showed that the high expression of LpCAT1 could be accompanied by an increase in the expression of matrix metalloproteinase (MMP-9). Accumulating studies confirmed that MMP-9 could facilitate the migration of tumor cells and cross-vascular invasion by dissolving extracellular matrix, thereby ultimately forming *in situ* and remote metastasis (26). It can also explain that HCC patients with an elevated expression of LpCAT1 were related to vascular invasion and advanced tumor staging. Moreover, Zhou et al. found that the expression of LpCat1 was positively correlated with the progression of prostate cancer (27). Another study showed that LpCat1 was expressed at least in normal breast tissues, relatively elevated in fibrocystic breast tissues, and most expressed in primary breast cancer tissues (8). Collectively, high level of LpCat1 promoted tumor progression, which might be a potential therapeutic target for HCC.

To understand the role of LpCat1 in the development of HCC, we evaluated the impact of altered levels of LpCat1 on the proliferation and metastasis of HCC *in vitro* and *in vivo*. Our data showed that the overexpression of LpCat1 enhanced viability and motility of HCC cells, while the down-regulation of LpCat1 inhibited cell proliferation, migration and invasion potential. Of note, with the knockdown of LpCat1, the number of lung nodules was decreased or even absent in metastasis model of mice, and the liver tumors of mice were smaller and lighter in xenograft model. These results indicated that down-regulation of LpCat1 effectively inhibited the growth and metastasis of HCC. Similarly, knockdown of LpCat1 showed not only cellular proliferation but also invasiveness and migration were both repressed in oral squamous cell carcinoma (9). Reduction of LpCat1 level in lung adenocarcinoma obviously restrained tumor growth and brain metastases *in vivo* (11). In clear cell renal cell carcinoma cells, depletion of LpCat1 decreased PCs, inhibited biological phenotype of cancer cells, and induced cell cycle arrest at G0/G1 phase (10). Furthermore, we investigated the effect of LpCat1 on cell cycle of HCC cells. LpCat1 accelerated cell cycle progression and knockdown of LpCat1 arrested cell cycle at G0/G1 phase in HCC cells, which was in consistent with the previous reported results.

To explore the underlying molecular mechanism of LpCat1 in HCC, we detected a series of cancer-related genes. The expression of cell cycle-related genes including CyclinD1, CyclinE and CDK4 were positively correlated with LpCat1 level in HCC cells. Moreover, LpCat1 could reduce the level of p27^kip1^, which was commonly recognized as a cell cycle inhibitor (28, 29). These results indicated that LpCat1 could accelerate cell cycle to maintain the aggressive proliferation of HCC cells by regulating the cell cycle-related genes expression. MMP-9, one of the cell metastatic markers (30), was found obviously increased in the LpCat1 over-expressed HCC cells and tissues. IHC analysis showed LpCat1 up-regulation increased ki67 expression in xenograft tumors. These findings suggested that LpCat1 promoted HCC cell proliferation, migration, invasion and cell cycle progression through activating several cancer-related genes. To clarify how LpCat1 affects these factors, we explored the proteins binding to LpCat1 in HCC cells. Based on IP-tandem LC/MS technology, we identified a variety of proteins, which were suspected to interact with LpCat1. Analyzing the results, STAT1 was predicted to be the interacting target with LpCat1, and we proved the direct interaction between STAT1 and LpCat1 in HCC cells by co-IP. As an important signal transduction factor, STAT1 was usually considered as a tumor suppressor in multiple cancers including HCC (31–34). Chen et al. reported STAT1 induced G0/G1 cell cycle arrest through reduction of cell cycle regulators including CyclinA, CyclinD1, CyclinE and CDK2 in HCC cells (35). In addition, researchers have also shown that the enhanced STAT1 expression in fibrosarcoma cells could restrain tumor formation and metastasis in mice by down-regulation of MMP-2 and MMP-9 (36). Consistent with our previous speculation, the present results demonstrated that up-regulation of STAT1 caused by LpCat1 knockdown inhibited cell proliferation, migration and invasion, arrested cell cycle at G0/G1 phase, accompanying by promotion of CyclinD1, CyclinE, CDK4 and MMP-9 levels, but a reduction of p27^kip1^ expression. In addition, we detected the expression of the phosphorylation state of STAT1 in Hep3B and Huh7 cells by western blotting, but got the negative results. STAT1 was predominantly present in unphosphorylated state in HCC cells, which had been confirmed by previous studies (37). Therefore, LpCat1 contributed to the development of HCC by directly interacting with STAT1.

In conclusion, we identified LpCat1 was significantly over-expressed in HCC tissues and cancer cells. Our study demonstrated that LpCat1 could directly interact with STAT1 and negatively regulate its expression level. High levels of LpCat1 not only promoted the expression of CyclinD1, CyclinE and CDK4, reduced the level of p27^kip1^ to accelerate the cell cycle process and cell proliferation, but also enhanced the cell migration and invasion by up-regulation of MMP-9. Conversely, STAT1 was released and increased in HCC cells with LpCat1 knockdown, contributing to cell cycle arrest at G0/G1 phase and inhibition of cell metastasis. Furthermore, what are the regulatory factors upstream of LpCat1? How to regulate? No relevant reports have been revealed so far, and we need to conduct in-depth research in the future. Overall, LpCat1 contributed to progressive HCC cell growth and metastasis. Knockdown of LpCat1 showed a strong inhibitory effect on the growth and metastasis of HCC *in vivo* and *in vitro*. In conclusion, LpCat1 might be a novel potential target for diagnosis and treatment of HCC.

## Data Availability Statement

The datasets presented in this study can be found in online repositories. The names of the repository/repositories and accession number(s) can be found in the article/**Supplementary Material**.

## Ethics Statement

The studies involving human participants were reviewed and approved by Committee on Ethics of Medicine, Navy Military Medical University (Shanghai, China). The patients/participants provided their written informed consent to participate in this study. The animal study was reviewed and approved by Animal Ethics Committee of Navy Military Medical University (Shanghai, China).

## Author Contributions

CS and MG conceived and designed this study. WJ, ZP, and BS performed the main experiments. LC assisted with the xenograft model construction in mice. QZ assisted with the statistical analysis of data. WJ wrote the original draft. ZP and BS reviewed and edited the manuscript. All authors contributed to the article and approved the submitted version.

## Funding

This study was financially supported by grants from the National Key Research and Development Program of China (No. 2018YFA0900900).

## Conflict of Interest

The authors declare that the research was conducted in the absence of any commercial or financial relationships that could be construed as a potential conflict of interest.

## Supplementary Material

Supplementary Figure 1 Cellular immunofluorescence was used to observe EGFP positive HCC cells at 72 h after infection with lentiviral vectors mediating LpCat1 overexpression or knockdown.

Supplementary Figure 2 qRT-PCR analysis of LpCat1 expression in HCC tissues and corresponding normal tissues (n=40).

Supplementary Figure 3 The expression level of STAT1 was detected by tissue microarray-based immunohistochemical analysis in HCC tissues. The representative images of STAT1 expression in HCC tissues and corresponding normal live tissues by IHC were taken, scale bar = 50 µm.
