# Supplementary figures and images for: LpCat1 Promotes Malignant Transformation of Hepatocellular Carcinoma Cells by Directly Suppressing STAT1
Source: Front Oncol. 2021 Jun 4;11:678714. doi: 10.3389/fonc.2021.678714 (PMC8220817; doi:10.3389/fonc.2021.678714)

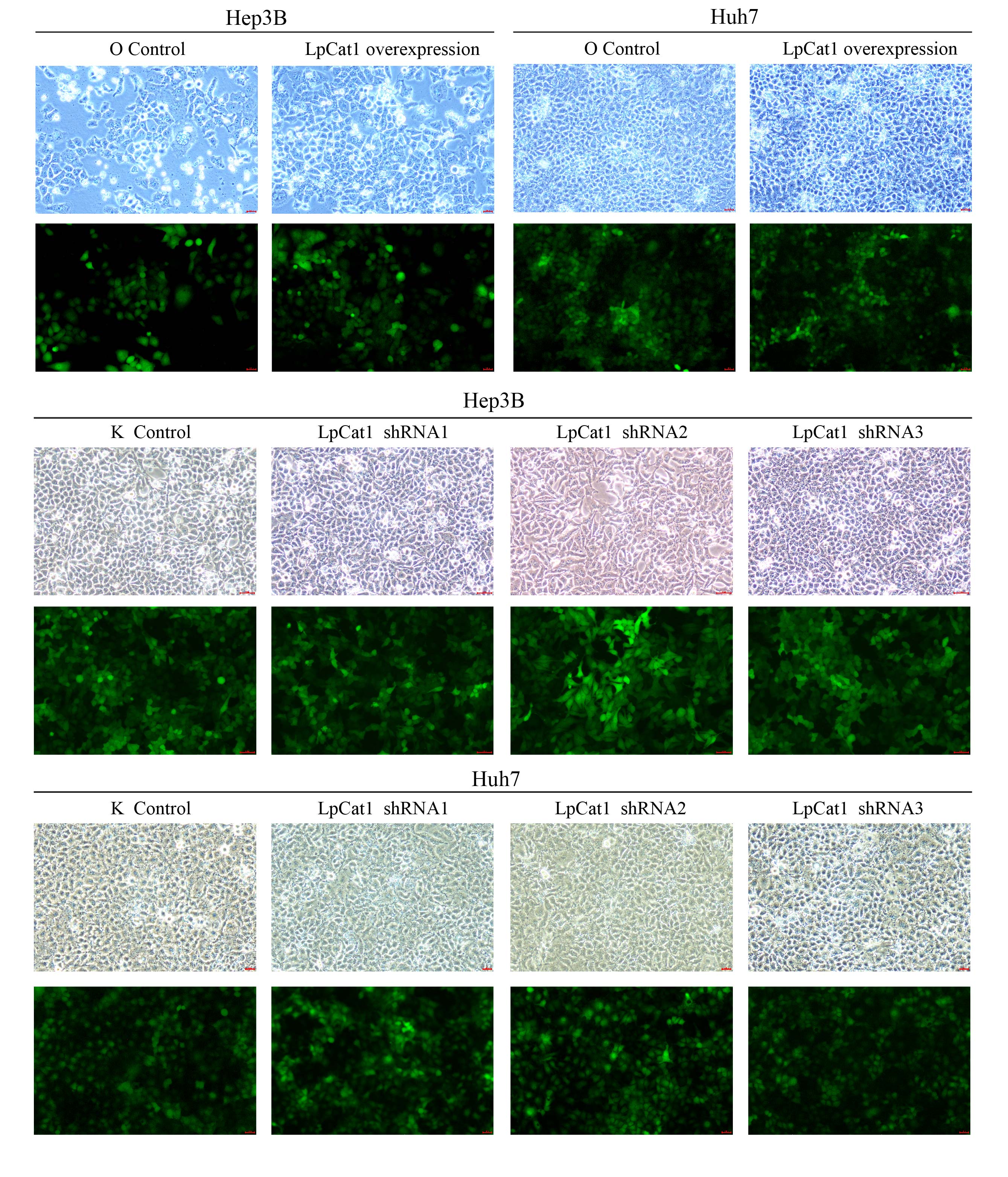

Supplement: Supplementary Figure 1 — Cellular immunofluorescence was used to observe EGFP positive HCC cells at 72 h after infection with lentiviral vectors mediating LpCat1 overexpression or knockdown. [file Image_1.jpeg]

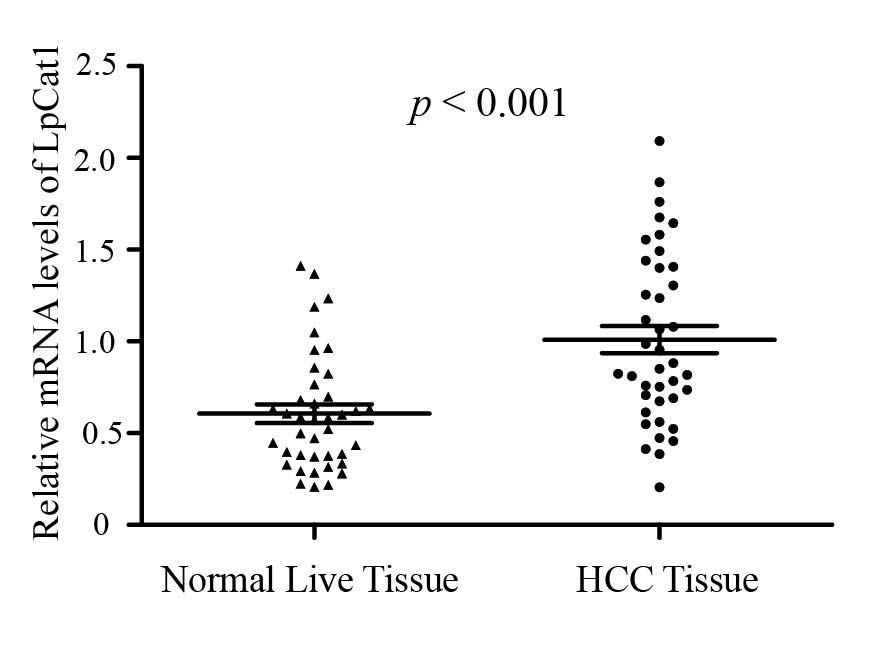

Supplement: Supplementary Figure 2 — qRT-PCR analysis of LpCat1 expression in HCC tissues and corresponding normal tissues (n=40). [file Image_2.jpeg]

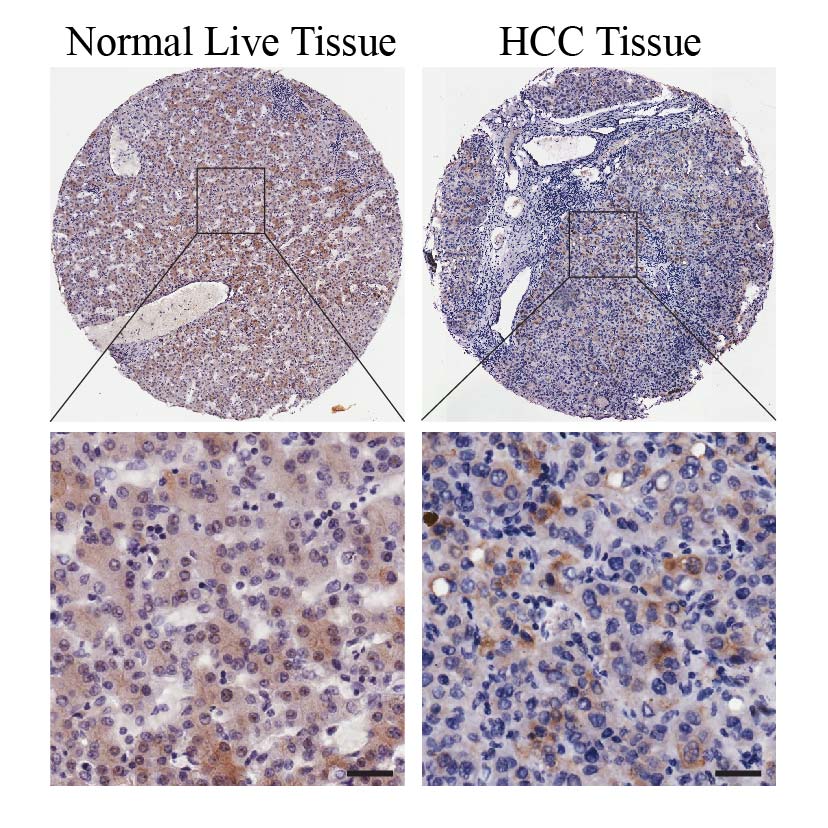

Supplement: Supplementary Figure 3 — The expression level of STAT1 was detected by tissue microarray-based immunohistochemical analysis in HCC tissues. The representative images of STAT1 expression in HCC tissues and corresponding normal live tissues by IHC were taken, scale bar = 50 µm. [file Image_3.jpeg]
