# Supplementary material for: LpCat1 Promotes Malignant Transformation of Hepatocellular Carcinoma Cells by Directly Suppressing STAT1
Source: Front Oncol. 2021 Jun 4;11:678714. doi: 10.3389/fonc.2021.678714 (PMC8220817; doi:10.3389/fonc.2021.678714)
Supplement: Supplementary file 4 [file Table_1.docx]

Table S1 The Sequences of LpCat1 shRNAs mediated by lentiviral vectors

| Name | Sequences (5’ to 3’) |
| --- | --- |
| Scramble shRNA | CCTAAGGTTAAGTCGCCCTCG |
| LpCat11 shRNA1 | GGAACTCTGATCCAGTATATA |
| LpCat11 shRNA2 | ACGGAAAGTGGCCACAGATAA |
| LpCat11 shRNA3 | AGATAGGTATTGCGGAGTTTG |
