# Supplementary material for: LpCat1 Promotes Malignant Transformation of Hepatocellular Carcinoma Cells by Directly Suppressing STAT1
Source: Front Oncol. 2021 Jun 4;11:678714. doi: 10.3389/fonc.2021.678714 (PMC8220817; doi:10.3389/fonc.2021.678714)
Supplement: Supplementary file 5 [file Table_2.docx]

Table S2 The primer sequences of LpCat1 and GAPDH

| Gene Name | The sequence of primers (5’ to 3’) |
| --- | --- |
| LpCat1 | Forward: TTACCTTCAAACCTGGTGCATT |
|  | Reverse: CGTGAGCCACAGGATTTCC |
| GAPDH | Forward: CATCTCTGCCCCCTCTGCTGA |
|  | Reverse: GGATGACCTTGCCCACAGCCT |
