# Supplementary material for: LpCat1 Promotes Malignant Transformation of Hepatocellular Carcinoma Cells by Directly Suppressing STAT1
Source: Front Oncol. 2021 Jun 4;11:678714. doi: 10.3389/fonc.2021.678714 (PMC8220817; doi:10.3389/fonc.2021.678714)
Supplement: Supplementary file 6 [file Table_3.docx]

Table S3 Antibodies used in western blot and IHC analysis

| Name of Antibodies | Catalog Number |
| --- | --- |
| Anti-LpCat1 Antibody | Proteintech 16112-1-AP |
| Anti-STAT1 Antibody | Proteintech 10144-2-AP |
| Anti-Cyclin D1 Antibody | Cell Signaling Technology #55506 |
| Anti-Cyclin E Antibody | Cell Signaling Technology #20808 |
| Anti-CDK4 Antibody | Cell Signaling Technology #12790 |
| Anti-p27^kip1^ Antibody | Abcam ab32034 |
| Anti-MMP-9 Antibody | Cell Signaling Technology #3852 |
| Anti-ki67 Antibody | Cohesion CQA3683 |
