# Supplementary material for: LpCat1 Promotes Malignant Transformation of Hepatocellular Carcinoma Cells by Directly Suppressing STAT1
Source: Front Oncol. 2021 Jun 4;11:678714. doi: 10.3389/fonc.2021.678714 (PMC8220817; doi:10.3389/fonc.2021.678714)
Supplement: Supplementary file 7 [file Table_4.docx]

**Table S4 Clinical characteristics of HCC patients according to the expression of LpCat1.**

| Variables | LpCat1 expression | | *P* value |
| --- | --- | --- | --- |
|  | **Low** | **High** |  |
|  | **( n = 27)** | **( n = 63 )** |  |
| Sex | | | |
| Female | 10 | 24 | 0.924 |
| Male | 17 | 39 |  |
| Age (years) | | | |
| $\boldsymbol{\geq}$50 | 16 | 35 | 0.745 |
| $\mathbf{<}$50  HBV status | 11 | 28 |  |
| Yes | 18 | 38 | 0.569 |
| No | 9 | 25 |  |
| Cirrhosis | | | |
| Yes | 8 | 30 | 0.113 |
| No | 19 | 33 |  |
| AFP (ng/mL) | | | |
| $\mathbf{>}$20 | 21 | 46 | 0.635 |
| $\boldsymbol{\leq}$20  Tumor size (cm) | 6 | 17 |  |
| $\mathbf{>}$5 | 12 | 22 | 0.249 |
| $\boldsymbol{\leq}$5  Tumor number | 15 | 41 |  |
| Multiple | 7 | 24 | 0.266 |
| Single | 20 | 39 |  |
| Tumor encapsulation | | | |
| Yes | 18 | 29 | 0.073 |
| No | 9 | 34 |  |
| Vascular invasion | | | |
| Yes | 10 | 45 | 0.002 |
| No | 17 | 18 |  |
| TNM stage | | | |
| I-II | 14 | 16 | 0.015 |
| III-IV | 13 | 47 |  |

HBV, hepatitis B virus; AFP, α-fetoprotein; TNM, tumor lymph node metastasis.

p < 0.05 was considered statistically significant.
